# Global Detection of Live Virtual Machine Migration Based on Cellular Neural Networks

**DOI:** 10.1155/2014/829614

**Published:** 2014-05-06

**Authors:** Kang Xie, Yixian Yang, Ling Zhang, Maohua Jing, Yang Xin, Zhongxian Li

**Affiliations:** ^1^College of Information Science and Engineering, Shandong University, Jinan 250100, China; ^2^Information Security Center, Beijing University of Posts and Telecommunications, Beijing 100876, China; ^3^Northeastern University & College of Information Science and Engineering, Shenyang 110819, China; ^4^National Cybernet Security Ltd., Beijing 100088, China

## Abstract

In order to meet the demands of operation monitoring of large scale, autoscaling, and heterogeneous virtual resources in the existing cloud computing, a new method of live virtual machine (VM) migration detection algorithm based on the cellular neural networks (CNNs), is presented. Through analyzing the detection process, the parameter relationship of CNN is mapped as an optimization problem, in which improved particle swarm optimization algorithm based on bubble sort is used to solve the problem. Experimental results demonstrate that the proposed method can display the VM migration processing intuitively. Compared with the best fit heuristic algorithm, this approach reduces the processing time, and emerging evidence has indicated that this new approach is affordable to parallelism and analog very large scale integration (VLSI) implementation allowing the VM migration detection to be performed better.

## 1. Introduction

Cloud computing provides ability to dynamically scale or shrink the provisioned resources as per the dynamic requirements [1]. It is elastic in nature and works on pay as its clients use model. Virtualization as a key concept of cloud computing gives an abstract view of hardware by means of multiple virtual guest operating systems which are known as Virtual machines (VMs) and are instanced on a single physical machine (PM). That is, depending on the capacity of the PM, many VMs can be created on it. It is also clear that the proper management of VMs can improve the resource utilization efficiently and carry out the isolation to multitenancy. Virtual machine monitor (VMM), which is also known as hypervisor, is simply a software and can be used as an interface between PM and VMs. One of the widely used VMM is Xen which allows various VMs to share common hardware in a safe and resource managed condition but without sacrificing the performance [2].

VM migration is a key enabler for dynamic resource management in cloud-based systems. Live VM migration as an extremely powerful tool for the cloud managers transfers state of a VM from one PM to another and can mitigate overload conditions and enables uninterrupted maintenance activities. In the migration process, complete state of running VM, which includes the permanent storage (i.e., the disk image), volatile storage (the memory), the state of connected devices (such as network interface card), and the internal state of the virtual CPU (VCPU), has to be transferred [2]. In this case, VM locations are varied dynamically and the internal state of the VCPU and connected devices are a few kilobytes of data and can be easily sent to the VMM and the target PM. For these reasons, it is clear that VMs can be migrated from overloaded PM to underloaded PM, but it will be only helpful when the efficient live migration techniques are used. Thus, we need an approach to trace and detect the processes migration in VM and mapping relationship between VCPUs and PM from the hypervisor's point of view, in order to verify whether the new scheduler is effective and the result is consistent with the initial idea.

Therefore, to meet the demands of processing time-saving, a lightweight algorithm of live VM migration detection (VMMD) based on cellular neural network (CNN) is proposed in this paper. CNN has features with multidimensional array of neurons and realizable paradigm of parallel computation; the processing time is unrelated with the data dimension. Moreover, it can be implemented by very large scale integration (VLSI), which makes neural networks easily implemented [3]. Therefore, it has been widely used in many fields [3–7], such as the classification and recognition of moving targets [4, 5], path detection of mazes [6], and microarray image reconstruction [7]. To the best of our knowledge, few reports have been published on the application in live VM migration detection.

The rest of this paper is organized as follows.  Section 2 provides an overview of the most adopted VM migration techniques. In Section 3, the proposed algorithm of live VMMD based on CNN is discussed.  Section 4 explains the details for designing CNN parameters via bubble sort particle swarm optimization (BSPSO) algorithm. After that, Section 5 presents the experimental results, followed by the conclusions in Section 6.

## 2. Related Works

In the proposed VM migration policy, VM migration is triggered when the data transfer time crosses a certain threshold due to the unstable network. Here, the threshold can be determined by a time-related service level agreement (SLA) between the cloud facility provider and the cloud user [8]. To solve the problem of host overload detection, a novel approach based on a Markov chain model is proposed by maximizing the mean intermigration time under the specified quality of service (QoS) goal [9]. In [10, 11], migration strategies are generally based on the CPU utilization, and when CPU utilization of a host exceeds a certain threshold, tasks implementing on the VM with the highest CPU utilization on the host will be migrated out. These strategies are generally lack of resources sensitivity and can not realize the dynamic resource allocation according to the actual conditions of the load.

In [12], Celesti et al. propose a composed image cloning (CIC) methodology able to reduce the cost of the VM disk-image relocation over a WAN. In [13], a new live VM migration strategy is proposed by using the load characteristics to implement hotspots detection, selection of VM, and migration destination host according to some multithreshold patterns. Srikantaiah models the migration problem as a modified bin packing problem in [14]. Firstly, the optimal point is determined from profiling data. Then, the heuristic algorithm is used to maximize the sum of the Euclidean distances of the current allocations to the optimal point at each server. Luo et al. [15] propose an advanced VM dynamic consolidation system through live migration approach. The system consists of three main components: load monitor which collects resources usage statistics from each server node, relocation planner which calculates the relocation plan by analyzing the resource usage histories, and VM controller which activates live migration to server nodes according to the results from relocation planner. In [16], Li et al. propose a live migration strategy for VM, which combines with performance predicting algorithm. According to the average utilization of the CPU, memory, I/O, and network bandwidth, the proposed strategy makes a serial of judgments about migration, such as whether a migration is triggered, what VM should be migrated, and where is the destination PM of the VM. But these existing researches are generally heuristic based or heavily rely on statistical analysis of historical data; thus, the optimization procedure has to be conducted after a relatively long period to obtain statistics.

To trace the migration process in VM, Zhang et al. [17] present a demo process migration tracing engine for monitoring the migration of process on VCPUs and VCPUs on the cores of physical processor based on Linux 2.6 and Xen 3.2. In [18], Liu et al. design a novel approach CR/TR-Motion that adopts recovery and trace technology to provide fast, transparent VM migration. With execution trace logged on the source PM, a synchronization algorithm is performed to orchestrate the running source and target VM until they get a consistent state. According to the above issue, it is clear that it is important for the proper management of virtualization resources information and security events, monitoring and analysis of VM situations, and migration policies. Therefore, we study the tracing method for history of infected VMs following the analysis of the VM migration lifecycle status and running states.

## 3. The Processing of VMMD Based on CNN

CNN is a large scale nonlinear locally connected analog circuit which processes signal in real time that was first proposed by the Professor Chua and Dr. Yang who came from the University of California in 1988 [3]. A CNN is composed of basic processing units called cells. Each cell, denoted by *c*(*i*, *j*), is connected to its neighboring ones; therefore, only the adjacent cells can directly connect with each other, others interaction are through dynamic continuous propagation effects. Each cell has the same structure, composed by a small amount of linear and nonlinear circuit elements.

The circuit equations of a cell which satisfy the KCL and KVL are easily derived as follows.

State equation is

(1) Cdvxij(t)dt=−1Rvxij(t)+∑c(k,l)∈Nr(i,j)A(i,j;k,l)vykl(t) +∑c(k,l)∈Nr(i,j)B(i,j;k,l)vukl(t)+I.

Output equation is

(2) $$\begin{array}{c} v_{ykl}\left(t\right)=f\left(v_{xij}\right)=\frac{1}{2}\left(\left|v_{xij}\left(t\right)+1\right|-\left|v_{xij}\left(t\right)-1\right|\right). \end{array}$$

Constraint conditions are

(3) $$\begin{array}{c} \left|v_{xij}\left(0\right)\right|\leq 1,\;\;\left|v_{uij}\left(0\right)\right|\leq 1, \end{array}$$

where 1 ≤ *i* ≤ *M*, 1 ≤ *j* ≤ *N*. The dynamics of a CNN has both output feedback and input control mechanisms. The output feedback effect depends on the interactive parameter *A*(*i*, *j*; *k*, *l*) and the input control effect depends on *B*(*i*, *j*; *k*, *l*). The key of CNN-based solution is to design the appropriate parameters of the feedback matrix *A* and control matrix *B* which represent the connection weight between cells.

Virtualization provides virtualized view of resources used to instantiate VMs. Once a VM is instantiated, a resource monitoring engine which is called VMM or hypervisor will track the resource usage and performance indicators related to the applications of the VM; it also manages and multiplexes access to the physical resources, maintaining isolation between VMs at all times.

Let VM_*n*_ = *vm*_*n*_^1^ ∪ *vm*_*n*_^2^ ∪ ⋯*vm*_*n*_^*m*^ denote the static state matrix of VM numbered *n*, composed of the union of *m* continue time state components *vm*_*n*_^1^, *vm*_*n*_^2^,…, and *vm*_*n*_^*m*^. The allocation matrix of VM *P*_*g*_ = {*P*_11_,…, *P*_*i*,*j*_,…, *P*_*l*,*s*_} can be divided into the state set of VM *V* = {VM_1_,…, VM_*l*_} and physical host (PH) *H* = {*H*_1_,…, *H*_*s*_}, if a VM numbered *v* is allocated into the host named *h*, *P*_*hv*_ = 1; otherwise, *P*_*hv*_ = −1. Any allocate solutions must meet the capacity constraints of PHs as follows:

(4) $$\begin{array}{c} \forall h\in \left\{1,\ldots ,n\right\}\;\overset{l}{\underset{v=1}{\sum }}P_{hv}\times \text{CPU}\left(V_{v}\right)\leq \text{CPU}\left(H_{h}\right),\\ \forall h\in \left\{1,\ldots ,n\right\}\;\overset{l}{\underset{v=1}{\sum }}P_{hv}\times \text{Mem}\left(V_{v}\right)\leq \text{Mem}\left(H_{h}\right), \end{array}$$

where the idle host will be closed to save energy.

Let VM_0_ denote the VMM which is called triggering cells, and let VM′ = VM∖VM_0_ as the initial state denote the remainder pattern, to be deleted from VM. VM′ is applied as input to the global state detection of VM by the cloning templates given by (5) [3]. Since VM′ ∈ VM, each state of VM at the initial time *t* = 0 can assume the three combinations of (input, initial  state) = (*u*_*i*,*j*_, *x*_*i*,*j*_(0)); namely, (VM_suspend_, VM_suspend_) = (−1, −1), (VM_copy_, VM_suspend_) = (1, −1), (VM_copy_, VM_copy_) = (1,1). Consider

(5) $$\begin{array}{c} A=\left[\begin{array}{ccc} 0 & \frac{c}{2} & 0\\ \frac{c}{2} & a & \frac{c}{2}\\ 0 & \frac{c}{2} & 0 \end{array}\right],\;\;B=\left[\begin{array}{ccc} 0 & -\frac{c}{2} & 0\\ -\frac{c}{2} & b & -\frac{c}{2}\\ 0 & -\frac{c}{2} & 0 \end{array}\right],\\ Z=z, \end{array}$$

where *a*, *b*, *c*, and *z* are real numbers and *c* > 0.

The steady state output *y*_*i*,*j*_(*∞*) of each cell *P*_*i*,*j*_ belonging to any connected component obeys two static local rules and one dynamic global rule:

*Local Rule 1*. If *z* ≤ *a* + *b* − 1, (*u*_*i*,*j*_, *x*_*i*,*j*_(0)) = (−1, −1), thus *y*_*i*,*j*_(*∞*) = −1, independent of (*u*_*k*,*r*_, *x*_*k*,*r*_(0)) of its neighbors states.

*Local Rule 2*. If *z* ≥ 1 − *a* − *b*, (*u*_*i*,*j*_, *x*_*i*,*j*_(0)) = (1, −1), thus *y*_*i*,*j*_(*∞*) = 1, independent of (*u*_*k*,*r*_, *x*_*k*,*r*_(0)) of its neighbors states.

**Proof**

Consider

(6) xi,j•(t)=−xi,j(t)+ayi,j(t)+bui,j +c2∑(k,r)∈I[yi+k,j+r(t)−ui+k,j+r(t)]+z=−xi,j(t)+ayi,j(t)+wi,j(t),

where *i* = 1,2,…, *l*, *j* = 1,2,…, *s*.

Assume that the parameters satisfy *a* > 1/*R*_*x*_ = 1, and then each cell of the CNN must settle at a stable equilibrium point after the transient has decayed to zero, and lim⁡_*t*→*∞*_⁡*v*_*yij*_(*t*) = ±1, 1 ≤ *i* ≤ *M*, 1 ≤ *j* ≤ *N* guarantee that our CNNs have binary-value outputs. This property is very important for detection problems in live VM migration.

**Figure 1:**
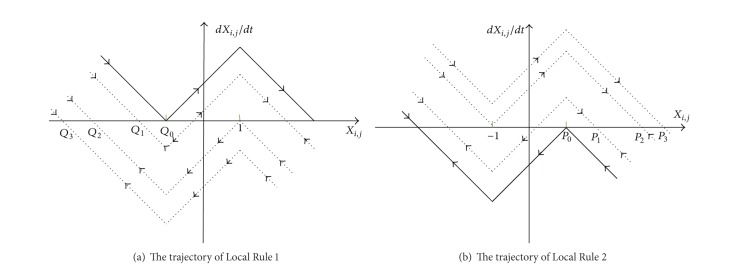
The dynamic trajectory of cell for different local rules.

(i) If (*u*_*i*,*j*_, *x*_*i*,*j*_(0)) = (−1, −1), in order to guarantee Local Rule 1 to be hold, *y*_*i*,*j*_(*∞*) = −1. From Figure 1(a), *x*_*i*,*j*_^•^ ≤ 0, the dynamic trajectory of cell *P*_*i*,*j*_ must tend to an equilibrium *Q*_0_ = −1, and the following inequality must be satisfied.

Thus,

(7) $$\begin{array}{c} 1-a+w_{i,j}\left(t\right)\leq 0,\\ z\leq a+b-1. \end{array}$$

(ii) If (*u*_*i*,*j*_, *x*_*i*,*j*_(0)) = (1, −1), in order to guarantee Local Rule 2 to be hold, *y*_*i*,*j*_(*∞*) = 1. From Figure 1(b), *x*_*i*,*j*_^•^ ≥ 0, and the dynamic route of cell *P*_*i*,*j*_ is not below the curve with *w*_*i*,*j*_ = 1 − *a* and finally tends to an equilibrium *P*_0_ = 1; the following inequality must be satisfied:

(8) $$\begin{array}{c} a-1+w_{i,j}\left(t\right)\geq 0. \end{array}$$

Thus,

(9) $$\begin{array}{c} z\geq 1-a-b. \end{array}$$

*Global Rule*. If *z* > −*a* − *b* − 2*c* − 1, (*u*_*i*,*j*_, *x*_*i*,*j*_(0)) = (1,1), and there exists an adjacent triggering cell or a directional connected path defined by I from cell *P*_*i*,*j*_ to some marked cell *P*_i*,j*_, *y*_*i*,*j*_(*∞*) = 1. Otherwise, *z* < 1 − *a* − *b* + 2*c*, *y*_*i*,*j*_(*∞*) = −1.

**Proof**

If there exists a directional connected path from cell *P*_*i*,*j*_ to some marked cell *P*_i*,j*_, then there exists some connected path from cell *P*_*i*,*j*_ to some cell *P*_*i*′,*j*′_ and there is at least one adjacent triggering cell *P*_*i*′,*j*′_ connected to cell *P*_i*,j*_, (*u*_*i*,*j*_, *x*_*i*,*j*_(0)) = (1,1) and *y*_*i*,*j*_(*∞*) = −1. In this case, *x*_*i*,*j*_^•^ > 0.

Thus,

(10) $$\begin{array}{c} 1+a+w_{i,j}\left(t\right)>0, \end{array}$$

(11) $$\begin{array}{c} z>-a-b-2c-1. \end{array}$$

**Figure 2:**
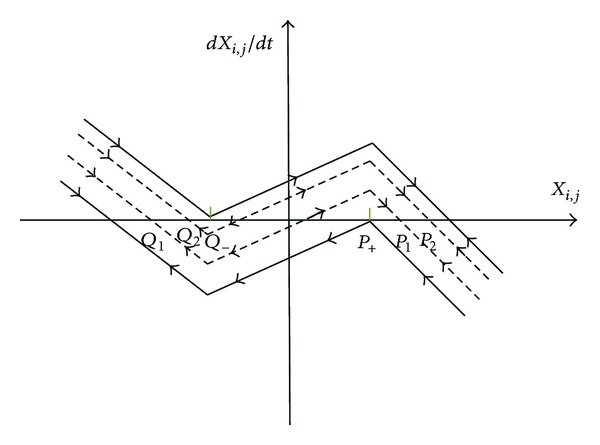
The dynamic trajectory of cell for global rule.

If there neither exit an adjacent triggering cell or a directional connected path defined by I from cell *P*_*i*,*j*_ to some marked cell *P*_i*,j*_, (*u*_*i*,*j*_, *x*_*i*,*j*_(0)) = (1,1). The dynamic trajectory of cell must tend to an equilibrium *P*_+_ indicated in Figure 2; that is, *x*_*i*,*j*_^•^ < 0.

Hence,

(12) $$\begin{array}{c} 1+a+w_{i,j}\left(t\right)<0, \end{array}$$

(13) $$\begin{array}{c} z<-1-a-b+2c. \end{array}$$

If (11) and (13) are satisfied, then the global rule holds. In summary, we complete the proof of Local Rules 1 and 2 and global rule.

## 4. The Design of CNN Template Parameters Based on BSPSO Algorithm

CNN is a typical nonlinear dynamic system with ability of optimization. The Lyapunov function, *E*(*t*), of a CNN by the scalar function is defined as follows:

(14) E(t)=−12∑(i,j)∑ (k,l)A(i,j;k,l)vyij(t)vykl(t) +12Rx∑(i,j)vyij(t)2−∑(i,j)∑ (k,l)B(i,j;k,l)vyij(t)vukl −∑(i,j)Zvyij(t),

where 1 ≤ *i*, *k* ≤ *M*; 1 ≤ *j*, *l* ≤ *N*. And it is a monotone decreasing function, always converges to a local minimum, where the CNN produces the desired output. Thus, the detected problem of live VM migration based on CNN can be changed into constrained optimization problem (COP) as follows:

(15) min⁡ ∂ECNN(t)∂t=−xi,j(t)+∑k,l∈Ni,j(r)Ak,lyk,l(t)        +∑k,l∈Ni,j(r)Bk,luk,l+Zi,j,

(16)  st {z≤a+b−1z≥1−a−bz>−a−b−2c−1z<−1−a−b+2c.

Most existing algorithms for COP use the penalty function method to handle constrains, which depends strongly on the penalty parameter [19, 20]. PSO as a global search optimization algorithm has been widely used in COP [21]. The algorithm was inspired by the social behavior of a flock of birds when searching for food. The potential solutions are denoted as particles, fly in the search space exploring for better regions. In PSO, each particle has a current position and a velocity and the particle finds the best solution through exchanges information with other experienced particles.

Assume that a swarm *P*(*k*) is composed of *N* particles in the *D*-dimensional solution space, where the position vector of particle *i* is *X*_*i*_ = (*x*_*i*1_, *x*_*i*2_,…, *x*_*id*_) and *V*_*i*_ = (*v*_*i*1_, *v*_*i*2_,…, *v*_*id*_) denotes the velocity vector. *P*_*i*_ = (*p*_*i*1_, *p*_*i*2_,…, *p*_*id*_) is the optimal solution vector experienced, that is, the best fitness solution *p*_best_ found by particle *i*. In other words, *P*_*g*_ = (*p*_*g*1_, *p*_*g*2_,…, *p*_*gd*_) denotes the best fitness solution *g*_best_ found by all of particles. The velocity and position update equations of each particle are shown as follows:

(17) Vi(t+1)=ω∗Vi(t)+c1∗r1∗(pbest−Vi(t)) +c2∗r2∗(gbest−Vi(t)),Xi(t+1)=Xi(t)+Vi(t+1),

where *i* = 1,2,…, *d*. The inertia weight *ω* is a factor used to control the balance of the search algorithm exploitation. The acceleration coefficient *c*_1_ moderates the maximum step size toward the global best particle, while another coefficient *c*_2_ moderates the step size toward the best personal position of that particle. They are bounded by  0 < *c*_1_, *c*_2_ < 2. *r*_1_ and *r*_2_ are random value in the range of [0,1] and usually selected its range artificially to reduce the probability that *V*_*i*_(*t*) and *X*_*i*_(*t*) escape from the search space.

There are no selection, crossover, and mutation operations to train the parameters of neural network by using PSO. The algorithm is simple, quick convergence, and the training precision is high, but, when a particle finds the local optimal solution, it will stop searching in the solution space, while other particles will quickly move closer to this particle; therefore, the algorithm is easy to fall into premature convergence. In addition, when the algorithm gets into a local optimum, an infeasible solution replacing mechanism is given to improve the search capability in this paper.

The basic idea of the proposed algorithm was to put feasible solutions and infeasible solutions into two different containers, respectively. Let *G*_fs_ = {*x*_1_, *x*_2_,…, *x*_*n*1_} denote the set of feasible solution, and the set of infeasible solution is *G*_ifs_ = {*y*_1_, *y*_2_,…, *y*_*n*2_}, where *n*1 + *n*2 = *N*. *F*_fs_(*x*) and *F*_ifs_(*y*) are the fitness function of *G*_fs_ and *G*_ifs_, respectively. Considering that the global optimal solutions often locate on or near the boundary of the feasible region for many COPs, we choose some better solutions in *G*_ifs_ based on bubble sort algorithm (BS) and add to *G*_fs_ in order to improve the searching ability. According to this, the value of *F*_fs_(*x*) should be sorted from small to large based on BS algorithm; that is, after BS processing, in the ordered set of feasible solution *G*_fs_′ = {*x*_1_′, *x*_2_′,…, *x*_*n*1_′}, *x*_1_′ is the best fitness solution. Similarly, *y*_1_′ is the best fitness solution in *G*_ifs_′.

In the sort processing, the competitive choice follows these rules:

1. any feasible solution is better than the infeasible solution;
2. in two feasible solutions, it gets ahead whose value of *F*_fs_(*x*) is superior;
3. in two infeasible solutions, smaller degree of *F*_ifs_(*y*) is superior.

The processing of improved PSO based on BS algorithm is given as follows.

**Step 1**

In accordance with the restraint condition, the particles' velocity and position are to be initialized. Iteration time (IT) IT = 1.

**Step 2**

For each particle, calculate the current value of the fitness function *F*(*x*) from the formula (15).

**Step 3**

Compare *F*(*x*) which is current experienced with the best fitness function *p*_best_, and if current *F*(*x*) is smaller than *F*(*p*_best_), the particle will be set to *G*_fs_ and become the new *p*_best_. Otherwise, the particle will be set to *G*_ifs_. Then, IT = IT + 1.

**Step 4**

Update the velocity and position of the particles according to formulas (5) and (6).

**Step 5**

After *n* times of iteration, obtain some superior particles in *G*_ifs_′ according to *F*_ifs_(*y*) and BS algorithm.

**Step 6**

Put the choosing particles into *G*_fs_ and get the best fitness function *g*_best_ of current globally experienced in *G*_fs_ based on BS algorithm.

**Step 7**

Determine whether the algorithm met the rule of iteration times, if it satisfied the condition then go to Step 8; otherwise, return to Step 2.

**Step 8**

Output the value of parameters *a*, *b*, *c*, and *z*.

**Figure 3:**
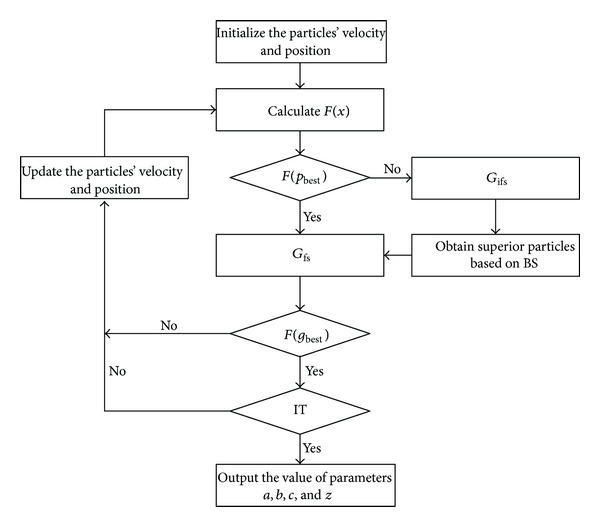
Flow diagram of BSPSO.

The flow diagram of BSPSO is as Figure 3.

## 5. The Simulation Results and Analysis

In this Section, we first introduce the performances of our BSPSO algorithm for the design of CNN template parameters, including the ability to get the template solution and the comparison results with GA, PSO BSPSO, and sort algorithm. Then, the performance of our VMMD model based on CNN algorithm is evaluated, such as the effects of VMMD model on migration lifecycle and the comparison results with other published existing algorithms.

### 5.1. The Experimentation Results of BSPSO Algorithm

In our study, we use MATLAB 7.6.0 software on the PC of 2 G memory to carry out this simulation experiments. In order to ensure stability and convergence of the BSPSO algorithm, accelerating factors *c*_1_ and *c*_2_ are made to 1.49, inertia factor is equal to 0.729, *r*_1_ and *r*_2_ are the random number in the interval [0,1], the initial state *v*_*xij*_(0) and *v*_*yij*_(0) of the randomly selected center cells are set to −1, other parameters *C* = 10^−9^ *F*, *R*_*x*_ = 1 *Ω*, and the maximum iterations is 2000 times. Finally, the algorithm gets the template solution as follows:

(18) $$\begin{array}{c} A=\left[\begin{array}{ccc} 0 & 0.64 & 0\\ 0.64 & 6.32 & 0.64\\ 0 & 0.64 & 0 \end{array}\right],\;\;B=\left[\begin{array}{ccc} 0 & -0.64 & 0\\ -0.64 & 2.19 & -0.64\\ 0 & -0.64 & 0 \end{array}\right],\\ Z=-7.36. \end{array}$$

**Figure 4:**
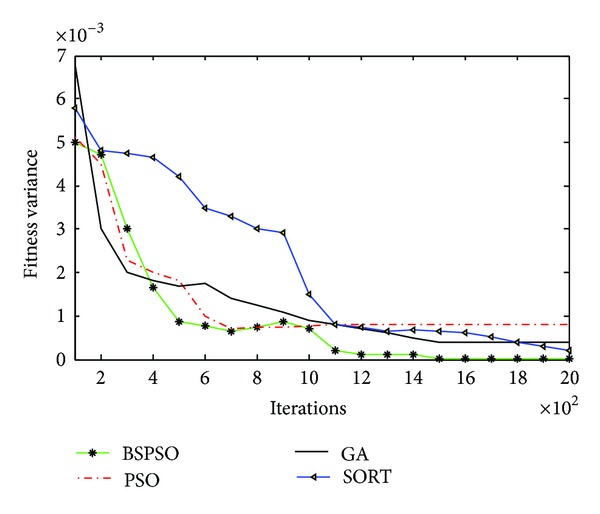
Simulation result of the optimal solution efficiency.

Then, we optimize the template parameter by using GA, PSO, BSPSO, and sort algorithm. In order to analyze the superiority of the proposed algorithm quantitatively, we define an evaluation criteria formula as (19) by using the number of iterations and fitness variance

(19) $$\begin{array}{c} \sigma^{2}=\overset{n}{\underset{i=1}{\sum }}\left(\frac{f_{i}-f_{\text{avg}}}{f}\right), \end{array}$$

where *f* is a normalization factor and can be taken as arbitrary values. *f*_*i*_ represents the fitness of the particle whose number is *i*, and *f*_avg_ denotes the average fitness of the particle swarm. The simulation result of the quantitative evaluation of the optimal solution efficiency is shown in Figure 4.

As depicted in Figure 4, the convergence speed of GA-based parameter optimization algorithm is faster than that of sort-based algorithm, and the performance of convergence speed of PSO algorithm is better than GA, but when the PSO algorithm finds a local optimal solution, it stops doing the searching, and sinks into the state of premature convergence. In the meantime, by adding the BS operating, the BSPSO model is slower than PSO in the first period of time, but it can jump out of the premature convergence and find the global optimum eventually.

### 5.2. The Experimentation Results of VMMD Based on CNN

This experimental environment contains four PHs, each host consists of Intel (R) Core (TM) 2Duo E8400@ 3.0 GHz, 16 GB memory and is divided into a number of VMs with different configurations. The PH4 acts as VMM and connected with PH1, 2, and 3 with NFS server by 1 Gbps network bandwidth line. The virtualization software is Xen4.1.2 based on Linux platform.

**Figure 5:**
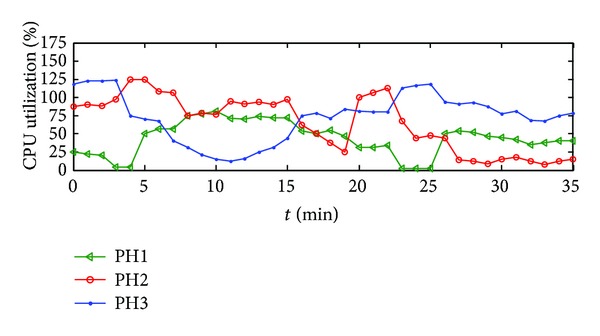
The sum of CPU percentage usage of VMs on each PH.

By “top” order, we obtain the extent of access to a resource, such as memory allocated or CPU allocated to a VM. In 35 minutes, the CPU percentage use of PHs is shown in Figure 5 and when the sum of CPU percentage use of VMs on each PH exceeds 100%, like PH1 and PH2, the VMs instantiated on that PH will migrate to other PMs because of the violation of SLA.

**Figure 6:**
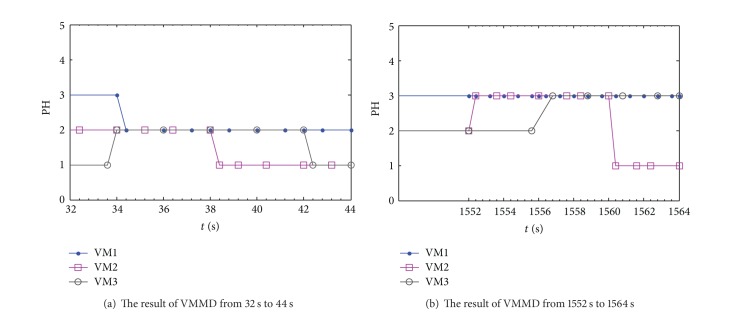
The result of VMMD based on CNN in different times.

By “public Map adjust VM Position (String umid)/∗@ param VM Name” order, we choose three VMs instantiated on different PH which are running EPA-HTTP benchmark tests, and the migration detection process based on CNN is shown in Figure 6.

As depicted in Figure 6(a), in the first period time, the state (memory pages) of VM3 is transferred from PH1 to PH2, and then the memory pages of VM1 migrate from PH3 to PH2; that is, VM1 and VM3 will suspend; they copy all its pages and then resume the VMs on the target machine PH2. The migration downtime is proportional to the size of the VMs and network resources available for state transfer. When the physical resources can not satisfy the need of VMs executed on PH2, each of which is self-contained with its own operating system, the targets of VM2 and VM3 will transfer from PH2 to PH1 in order and meet the sequence constraints. Similarly, Figure 6(b) also verifies the live VM migration policy satisfying the sequence constraints and SLA. The new PM can be added to offset the load of overloaded PM by migrating VM2 from PH3 to PH1. Likewise, hosting new VMs may result in future overloads of PH1, which will cause the migration requires to be triggered.

**Figure 7:**
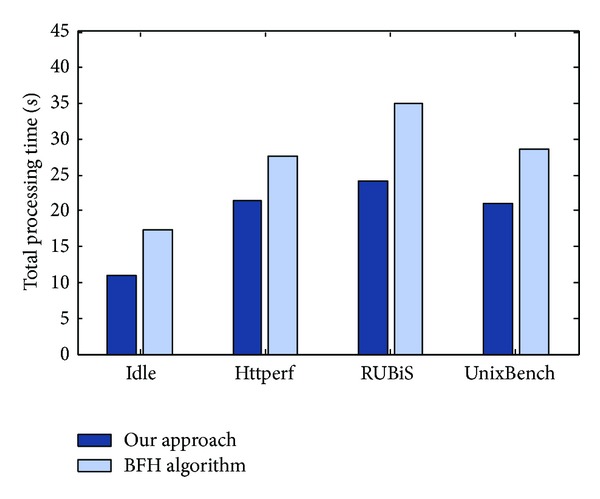
Total processing time of VMMD for different workloads.

Figure 7 shows the total processing time of VMMD between our approach and traditional best fit heuristic algorithm (BFH) [22] for different workloads in 30 minutes. Compared with BFH algorithm, our approach reduced the processing time by 36.78%, 22.1%, 30.86%, and 26.48% for the workload of dynamic application (Idle, Httperf, RUBiS, and UnixBench), respectively. The reason is that the migration policy based on BFH should determine when a PH is considered being underloaded; hence, it becomes a good candidate for hosting VMs that are being migrated from overloaded PHs, that cause the migration downtime of dynamic application to be extremely long. Whereas our detection approach only executes iterations to perform the process of the suspending and copy phase, it is a feasible method with satisfying lightweight performance and global live monitoring advantage.

## 6. Conclusions

In this paper, we have proposed a live VMMD model based on CNN, which can monitor the security of target operating systems and it also provides the ability to inspect the VMs' state. The migration procedure can be emerged as locally connected, nonlinear processor arrays, while the outputs reach their steady state values at an equilibrium point which represents a desirable feature in view of VLSI hardware implementations of real time networks. On the basis of analyzing the Local Rules 1 and 2 and the global rule, the parameter relationship can be mapped as a COP. Then, BSPSO algorithm has been designed to find out better optimum, avoiding the PSO algorithm trapping into local optimum. Experiments are carried out to demonstrate the performance of the proposed model, and the comparative results show that the proposed model exhibits superior performance with shorter processing time compared with BFH algorithm.
